# Bridging the Gap in Dementia Rehabilitation: Evaluating the INCLUDE Initiative's Impact on Allied Health Professionals’ Knowledge, Practices, and Advocacy

**DOI:** 10.1002/alz70858_097953

**Published:** 2025-12-24

**Authors:** Den‐Ching Angel Lee, Catherine Devanny, Keith Hill, Kate Swaffer, Grant Russell, Claire O'Connor, Natasha Layton, Alan Petersen, Barbara de‐Graff, Taya A Collyer, Terry Haines, Velandai K Srikanth, Michele L Callisaya

**Affiliations:** ^1^ National Centre for Healthy Ageing, Melbourne, VIC, Australia; ^2^ Rehabilitation, Ageing and Independent Living (RAIL) Research Centre, Frankston, VIC, Australia; ^3^ Monash University, Melbourne, VIC, Australia; ^4^ National Centre for Healthy Ageing, Frankston, VIC, Australia; ^5^ University of South Australia, Adelaide, SA, Australia; ^6^ Honorary Associate Fellow, Faculty of Science, Medicine & Health, University of Wollongong, Wollongong, NSW, Australia; ^7^ Co‐Founder and HR Advisor, Dementia Alliance International (DAI), Sydney, NSW, Australia; ^8^ Neuroscience Research Australia, Sydney, NSW, Australia; ^9^ School of Psychology, Faculty of Science, University of New South Wales, Sydney, NSW, Australia; ^10^ HammondCare, Sydney, NSW, Australia; ^11^ University of Tasmania, Hobart, TAS, Australia; ^12^ Peninsula Clinical School, Central Clinical School, Monash University, Melbourne, VIC, Australia; ^13^ Menzies Institute of Medical Research, University of Tasmania, Hobart, TAS, Australia; ^14^ Frankston Hospital, Peninsula Health, Melbourne, VIC, Australia; ^15^ Peninsula Health, Melbourne, VIC, Australia; ^16^ University of Tasmania, Hobart, Australia; ^17^ Menzies Institute for Medical Research, University of Tasmania, Hobart, TAS, Australia

## Abstract

**Background:**

Rehabilitation plays a crucial role in dementia care by addressing functional decline and enhancing quality of life. Despite growing evidence and international guidelines supporting rehabilitation, access remains limited for patients with dementia due to knowledge gaps and systemic barriers. The INCLUDE initiative was developed to address these challenges through interdisciplinary training and a Community of Practice (CoP) for allied health professionals (AHPs). This study aimed to evaluate the impact of the INCLUDE initiative, which includes online training modules and a CoP focussed on dementia rehabilitation, on AHPs' knowledge, attitudes, confidence, and practices.

**Method:**

A longitudinal pre‐post study design was employed. Group 1 (first intake) completed online modules and participated in CoP activities, while Group 2 (second intake) completed only the online modules. Data were collected at three time‐points: baseline (T1), post‐training (T2, 1‐2 months), and for Group 1 only, a 10‐month follow‐up (T3). Measures included the Dementia Attitude Scale (DAS), Dementia Knowledge Assessment Scale (DKAS), confidence ratings, and the Dementia Rehabilitation Scale Questionnaire. Social Network Analysis was used to map changes in professional connections within Group 1's CoP.

**Result:**

Preliminary analysis of combined data from Group 1 (*n* = 103) and Group 2 (*n* = 373) (*N* = 476) between T1 and T2 revealed significant improvements in dementia knowledge (DAS mean change: 3.5; *p* <0.001), social comfort (mean change: 6.9; *p* <0.001), and confidence in providing rehabilitation (mean change: 2.0; *p* <0.001). Additional insights from the T3 follow‐up with Group 1 are anticipated, offering further data on these measures with an emphasis on professional collaboration and workplace advocacy.

**Conclusion:**

The INCLUDE initiative enhances AHPs' knowledge, confidence, and interdisciplinary collaboration, addressing barriers to dementia rehabilitation. Anticipated T3 results will further illuminate the program's longer‐term impact on advocacy and practice change. This study highlights the value of ongoing education and peer collaboration in fostering inclusive dementia care.

